# Finding the right type of cell

**DOI:** 10.7554/eLife.86172

**Published:** 2023-02-16

**Authors:** Louis K Scheffer

**Affiliations:** 1 Janelia Research Campus, HHMI Ashburn United States

**Keywords:** machine learning, morphology, representation learning, 3D, cells, *P. dumerilii*

## Abstract

A new method allows researchers to automatically assign cells into different cell types and tissues, a step which is critical for understanding complex organisms.

**Related research article** Zinchenko V, Hugger J, Uhlmann V, Arendt D, Kreshuk A. 2023. MorphoFeatures for unsupervised exploration of cell types, tissues and organs in volume electron microscopy. *eLife* **12**:e80918. doi: 10.7554/eLife.80918.

Since the advent of microscopy in the 17^th^ century, it has become well established that organisms are divided into tissues made up of different types of cells, with cells of the same type typically performing the same role. This simplifies the task of understanding a biological system immensely, as there are many fewer cell types than individual cells (Masland, 2001).

Categorizing tissues and cell types has always been done manually, usually by grouping cells that look the same based on their shape, internal structures and various other features. This is also true for images collected using modern day techniques, such as electron microscopy, which can provide three-dimensional reconstructions of tissue samples, or even entire small organisms less than a millimeter cube in size.

While electron microscopy images can be automatically subdivided or ‘segmented’ into individual cells, assigning each one to a cell type by hand is both difficult and time consuming; for example, in a recent project, it took several experts many months to categorize one half of the fruit fly brain (Scheffer et al., 2020). The whole task becomes even more challenging if the object being studied is not a well-known model organism. Now, in eLife, Valentyna Zinchenko, Johannes Hugger, Virginie Uhlmann Detlev Arendt and Anna Kreshuk of the European Molecular Biology Laboratory report a new method that could simplify this process (Zinchenko et al., 2023).

Zinchenko et al. based their program on a machine learning method called unsupervised, contrastive learning (van den Oord et al., 2018). The program works by grouping cell types without having received prior examples of ‘human-classified’ cell types or features (i.e., it is unsupervised) and by finding features that maximize the difference (or contrast) between examples that should be grouped together and those that should not. The method requires many examples, both of cells that should be grouped together, and those that should not. For the positive examples (those that should be grouped together), Zinchenko et al. created synthetic copies of each existing cell with minor modifications, such as different rotations, reflections, and texture or shape variations. In this case, the original cell and the modified cell should be grouped together. For negative examples (those that are of different types), they picked pairs of cells at random from their sample. This will be wrong occasionally but it is sufficiently accurate to train their model while allowing unsupervised operation.

Machine learning was then applied to find features shared by the positive examples only. The system combined the learned features of each original cell into a vector that summarizes the cell’s shape and texture. The team found that cells belonging to the same type were close together within the space of the vector, which can be visualized and interpreted by existing dimension reduction techniques (McInnes et al., 2018).

Zinchenko et al. then tested their model on a three-dimensional reconstruction of the annelid worm (*Platynereis dumerilii*) obtained through electron microscopy. Their computer model was able to match the different cell types and could identify subgroups of cells that could not be distinguished using human-specified features. Moreover, when compared to a gene expression map of the whole animal, the cells that had been classified as similar based on their features also shared similar genetic signatures, more so than cells that had previously been clustered using ‘human-designed’ features.

Next, they extended their classification method to consider both the shape and texture of each cell, and a combination of these features of all physically adjacent cells. Grouping these enhanced features revealed different tissues and organs within the animal. The classification system of the model strongly agreed with human results, but also found subtle tissue distinctions and rare features that had been overlooked by humans examining the same data set. For example, the analyses revealed a specific type of neuron in the midgut region of the worm, which had previously not been confirmed to be located in this area of the body.

The ‘unsupervised’ aspect of the method created by Zinchenko et al. is critical because it means the program does not require a full library of the relevant cell types (or a full list of the features that can distinguish between the cell types). Instead, the program learns these characteristics from the data itself (Figure 1). This is particularly useful for systems where the cell types are not known. Moreover, it is not restricted to using cell features that humans deem important, such as the roundness of a cell or the presence of dark vesicles. This means that the model can often outperform humans and work without bias, as it is not told what to expect and is thus less likely to overlook rare or unexpected cell types.

**Figure 1.**
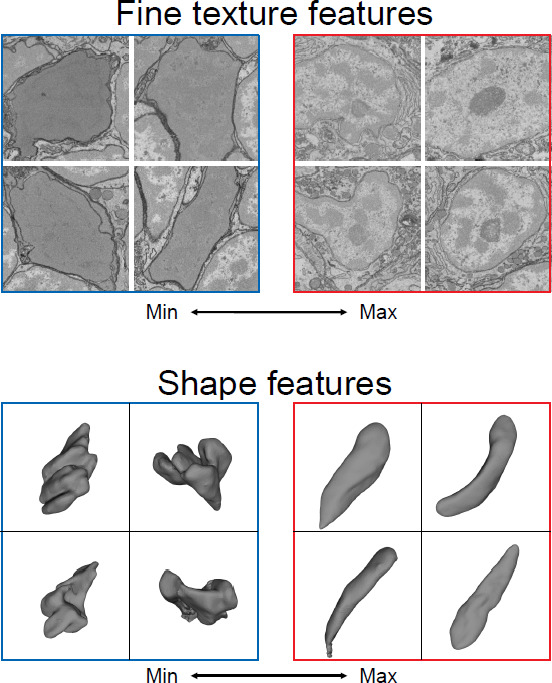
Making cell and tissue classification an automatic process. Three-dimensional reconstructions of organisms using electron microscopy harbor a multitude of scientific data about cell composition and structure. Zinchenko et al. created a machine learning system that can identify different features to distinguish one cell type from another. Since these features are learned, they do not necessarily correspond to human terms commonly used to describe the shape or texture of cells (such as rounded or speckled). The above images show cells with the best and worst match to two learned features, which can then be used to determine which aspect of the cell the feature corresponds to, such as texture (top) or shape (bottom).

Image credit: Adapted from Figure 3, Zinchenko et al., 2023 (CC BY 4.0).

Electron microscopy and related techniques provide an incredible level of detail, including the shape, location and structure of every cell. But analyzing this flood of data by hand is nearly impossible and automated techniques are desperately needed to unlock the potential of these findings (Eberle and Zeidler, 2018). Significant progress has already been made in turning some tasks, such as cell segmentation and identifying synapses, into automatic processes, leaving cell and tissue identification as some of the most time-consuming manual steps (Januszewski et al., 2018; Huang et al., 2018). By helping to automate this step, Zinchenko et al. make a critical step in the journey of understanding these invaluable but intimidating data sets.
